# Comparative Assessment of Various Cephalometric Parameters Used for Determining Vertical Skeletal Dysplasia

**DOI:** 10.7759/cureus.55101

**Published:** 2024-02-27

**Authors:** Pinaki Roy, Poulomi Roy, Sourav Koley

**Affiliations:** 1 Department of Orthodontics and Dentofacial Orthopedics, Burdwan Dental College and Hospital, Burdwan, IND; 2 Department of Dentistry, Maharaja Jitendra Narayan Medical College and Hospital, Cooch Behar, IND

**Keywords:** divergence, malocclusion, cephalometrics, growth pattern, vertical jaw relation

## Abstract

Introduction: The aim of the study is to assess and correlate the different cephalometric parameters used to determine the vertical jaw relationship.

Methods: Cephalometric radiographs from 148 patients were assessed and comparison was made using all eight parameters. Statistical analysis was performed using mean, standard deviation and coefficient of variance. A correlation was found between different variables using the Pearson's correlation coefficient.

Results: In the entire sample, the basal plane angle displayed the most variable distribution, while the R angle displayed the most homogeneous distribution. There was a significant correlation found between the Jarabak ratio, Steiner’s mandibular plane angle (SN-GoGn), Frankfort mandibular plane angle (FMA), R angle, and DR angle. There was a moderate to weak correlation between the Y-axis, basal plane angle, and facial height ratio with other skeletal analyses.

Conclusion: Among angular variables DR angle, R angle, SN-GoGn and FMA can be used and among linear variables Jarabak ratio could be used reliably to assess the growth pattern. It suggested that in order to obtain an accurate evaluation of the vertical jaw relationship, a variety of measurements should be combined.

## Introduction

Orthodontic diagnosis and treatment planning involves assessment of teeth, facial esthetics and proportions. Previously more attention was directed towards classifying malocclusion in antero-posterior or sagittal plane. Nonetheless, it has been recognized that the development of the human face is complex, necessitating the assessment and management of malocclusion in all three dimensions [1,2].

It has been noted that facial balance is highly influenced by the growth pattern in the vertical dimension. The excess growth in the vertical dimension may lead to gummy smile, open bite, incompetent lips and long face. Alternatively, deficient growth leads to reduced incisal display, overclosure of lips and short face [3]. Because the patient's vertical growth pattern affects several orthodontic treatment decisions, including anchorage requirements, macro-aesthetics, extraction vs. non-extraction, and surgical vs. non-surgical, evaluating the vertical jaw relationship is essential [4].

There have been reports of several cephalometric and non-cephalometric techniques for evaluating a patient's vertical pattern [5]. To evaluate the vertical jaw relationship, several angular and linear cephalometric methods have been employed; however, each has pros and cons of its own. There isn't a single, well-researched technique for figuring out discrepancy in the vertical plane in the literature [4,6,7]. The most often used angular parameters to evaluate vertical dysplasia are Steiner’s mandibular plane angle (SN-GoGn), Frankfort mandibular plane angle (FMA), basal plane angle, facial axis angle, and Y-axis. Popular linear measurements include the Jarabak ratio, sella (S)-gonion (Go), nasion (N)-anterior nasal spine (ANS), N-menton (Me), ANS-Me, and S-posterior nasal spine (PNS) [8]. Nonetheless, when evaluating vertical discrepancy, none of these parameters is absolute, and the same patient may have different values for each of them, making orthodontic diagnosis and treatment planning challenging. Thus, the goal of this study was to determine the correlation between the different cephalometric parameters use for evaluation of the vertical jaw relationship.

## Materials and methods

This is a cross-sectional retrospective study that was carried out in the Department of Orthodontics and Dentofacial Orthopedics, Government Dental College and Hospital, West Bengal after the approval from Institutional Ethics Committee. Lateral cephalogram of 148 subjects were selected for the study from the department's record archive. The study included subjects aged 16 to 28 who had never had orthodontic treatment before, had all of their permanent teeth up to their second molars, had no history of trauma, and no craniofacial disorders.

The imaging protocol for all selected radiographs was standard preset with Planmeca Proline EC machine (Helsinki, Finland) with the exposure values set at 68kVP and 12mA for a total of 0.5 seconds, using high-speed polyester based 18x24 cm Kodak X-Omat lateral head films (Rochester, NY, USA). Using the same X-ray machine and a standardized protocol, a single operator performed lateral standardized cephalograms, placing the patient in the natural head position in accordance with Solow and Tallgren's research [9]. An LED X-ray viewer was used to trace the lateral cephalogram on an A4 sheet of acetate paper using a 2B or 3HB hard lead pencil. A measuring scale was used to record the linear measurements with an accuracy of 0.5 mm. A protractor was used to analyse the angular measurements with an accuracy of 0.5°. From the cephalogram, the following parameters were calculated: Jarabak ratio, FMA, SN-GoGn, basal plane angle, Y-axis, R angle, DR angle, and facial height ratio.

**Figure 1:**
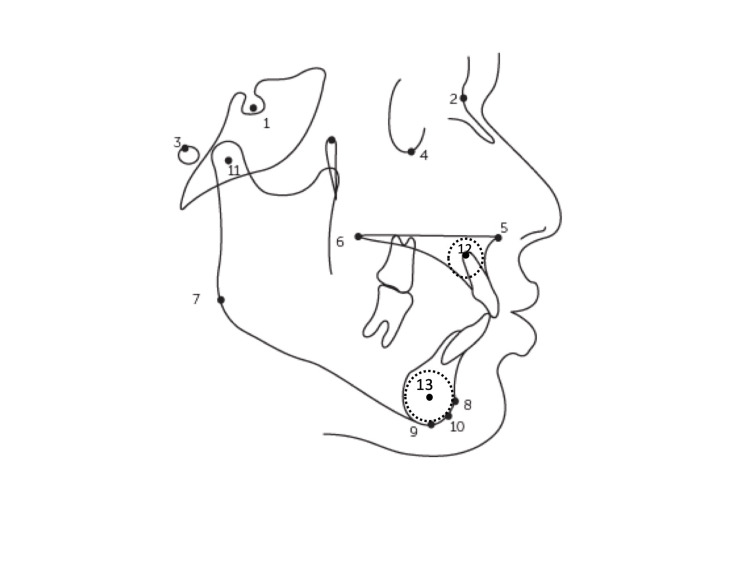
Skeletal landmarks 1. Sella (S): The midpoint of sella turcica. 2. Nasion (N): The most anterior point on the frontonasal suture. 3. Porion (Po): The posterosuperior margin of internal auditory meatus. 4. Orbitale (Or): The anteroinferior margin of orbital cavity. 5. Anterior nasal spine (ANS): The tip of anterior nasal spine of the palate. 6. Posterior nasal spine (PNS): The tip of posterior nasal spine at the junction of hard and soft palates. 7. Gonion (Go): The angle of mandible. 8. Pogonion (Pog): The most anterior point on bony chin. 9. Menton (Me): The most inferior point on bony chin. 10. Gnathion (Gn): The midpoint between pogonion and menton. 11. Condylion (Co): The center of the condyle head of the mandible. 12. Point M (M): Midpoint of premaxilla, obtained by forming the best fit circle that was tangent to the superior, anterior, and palatal surfaces of the maxilla and then approximating its center. 13. Point G (G): Center of the largest circle that is tangent to the internal inferior, anterior, and posterior surfaces of the mandibular symphysis. This figure is a creation of the authors of this paper.

**Figure 2:**
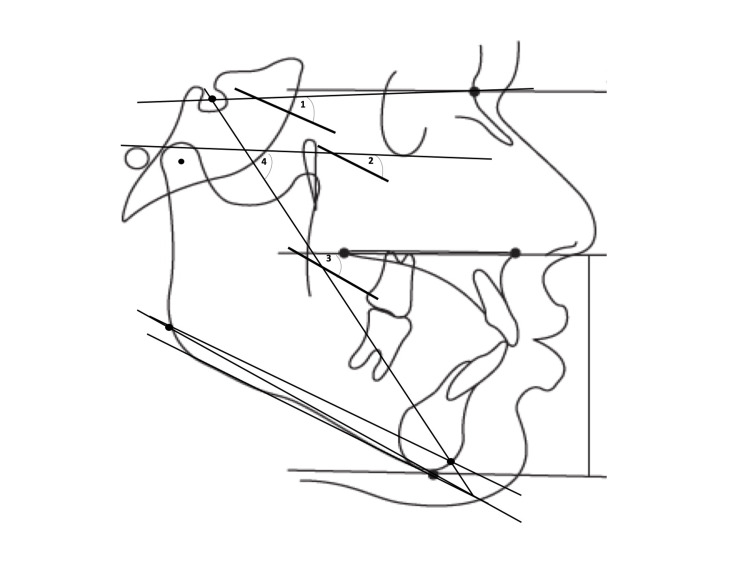
Vertical parameters 1. Steiner’s mandibular plane angle (SN-GoGn) (°) 2. Frankfort mandibular plane angle (FMA) (°) 3. Basal plane angle (°) 4. Y-axis angle (°) This figure is a creation of the authors of this paper.

**Figure 3:**
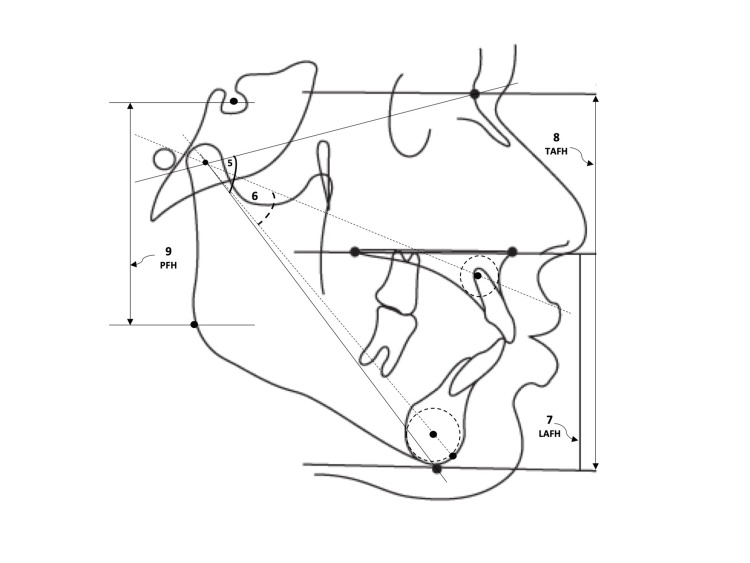
Vertical parameters (continued) 5. R angle (°) 6. DR angle (°) 7. Lower anterior facial height (LAFH): linear distance between ANS-Me 8. Total anterior facial height (TAFH): linear distance between N-Me 9. Posterior facial height (PFH): linear distance between S-Go ANS: anterior nasal spine, N: nasion, Me: menton, S: sella, Go: gonion This figure is a creation of the authors of this paper.

**Figure 4:**
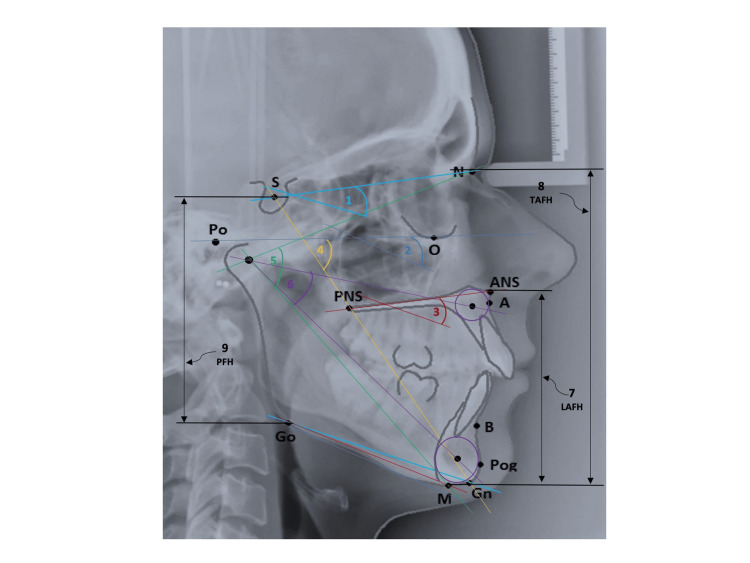
Vertical parameters marked in a lateral cephalogram 1. Steiner’s mandibular plane angle (SN-GoGn) 2. Frankfort mandibular plane angle (FMA) 3. Basal plane angle 4. Y-axis angle 5. R angle 6. DR angle 7. Lower anterior facial height (LAFH): linear distance between ANS-Me 8. Total anterior facial height (TAFH): linear distance between N-Me 9. Posterior facial height (PFH): linear distance between S-Go ANS: anterior nasal spine, N: nasion, Me: menton, S: sella, Go: gonion This figure is a creation of the authors of this paper.

The reference points, planes and angles used are shown in Table 1 and Figures 1-4.

**Table 1:** Cephalometric parameters used in the study LAFH: lower anterior facial height, PFH: posterior facial height, TAFH: total anterior facial height, ANS: anterior nasal spine, N: nasion, Co: condylion, Me: menton, S: sella, Go: gonion

Parameter	Description
1. SN-GoGn (°) (Steiner’s mandibular plane angle)	Angle between SN plane and Steiner’s mandibular plane
2. FMA (°) (Frankfort mandibular plane angle)	Angle between Frankfort horizontal (FH) plane and Tweed’s mandibular plane
3. Basal plane angle (°)	Angle between maxillary and mandibular plane
4. Y-axis angle (°)	Angle between sella-gnathion and FH plane
5. R angle (°)	Angle between N, Co and Me.
6. DR angle (°)	Angle uses three skeletal landmarks, the point C (centre of the condyle), point M (midpoint of premaxilla), and point G (centre of the largest circle that is tangent to the internal inferior, anterior, and posterior surfaces of the mandibular symphysis).
7. Facial height ratio [LAFH (mm)/TAFH (mm)]	Ratio between lower anterior facial height (ANS-Me) and total anterior facial height (N-Me)
8. Jarabak ratio [PFH (mm)/TAFH (mm)]	Ratio between posterior facial height (S-Go) and total anterior facial height (N-Me)

Method error

Dahlberg’s formula (mean error ratio) SE=d2/2n, where d=difference between the measurements at two different times; n=number of measurements) was used to determine the methodological error within the cephalometric analysis. Fifteen subject samples were randomly chosen and measurements of cephalograms were conducted, after a one-week interval by the same examiner.

Statistical analysis

The study's data were put through descriptive tests, and IBM SPSS software version 29.0.10 (IBM Corp., Armonk, NY, USA) was used to compute the mean, standard deviation, and coefficient of variation for each measurement. To determine whether the various vertical jaw parameters could be interchanged, the correlation between them was examined using Pearson’s correlation test.

## Results

**Figure 5:**
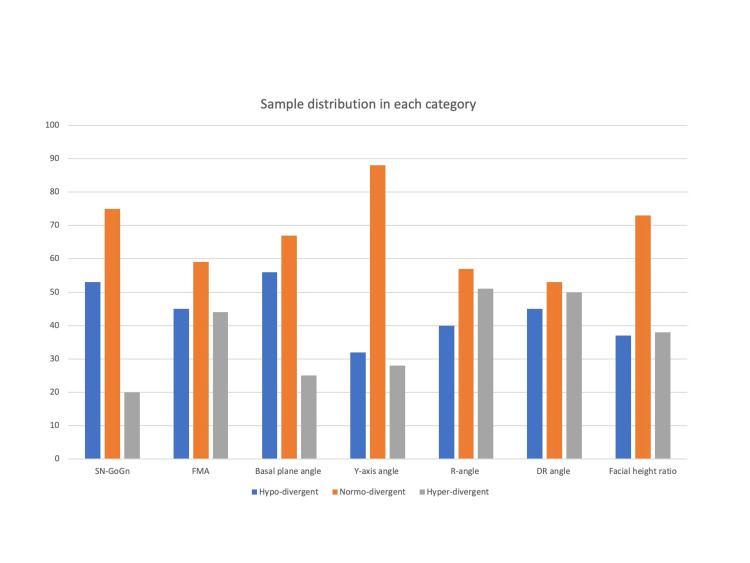
Graphical representation of sample distribution in each category. SN-GoGn: Steiner’s mandibular plane angle, FMA: Frankfort mandibular plane angle

The sample consisted of 148 subjects that included both females and males. The subjects were divided into hypo-divergent, normo-divergent and hyper-divergent groups (Table 2, Figure 5).

**Table 2:** Comparison of assessments of vertical jaw relationship

Parameters	No. of cases in each category		
	Hypo-divergent	Normo-divergent	Hyper-divergent
1. SN-GoGn (°) (Steiner’s mandibular plane angle)	53	75	20
2. FMA (°) (Frankfort mandibular plane angle)	45	59	44
3. Basal plane angle (°)	56	67	25
4. Y-axis angle (°)	32	48	68
5. R angle (°)	40	57	51
6. DR angle (°)	45	53	50
7. Facial height ratio	62	46	40
8. Jarabak ratio	37	73	38

Throughout the sample, class strata of Jarabak ratio, facial height ratio, R angle, DR angle, Y-axis angle, basal plane angle, FMA and SN-GoGn were defined in order to analyse the data. Eight cephalometric parameters were used to evaluate the vertical jaw relationship, and the results are displayed in Table 2 which shows the variations in case distribution within each skeletal group. Table 2 illustrates that of the eight indicators of the vertical skeletal relationship, parameters such as Jarabak ratio, Sn-GoGn and basal plane angle showed the highest percentage in the normo-divergent group; the facial height ratio showed the highest percentage in the hypo-divergent group, and the Y-axis angle showed the highest percentage in the hyper-divergent group.

The means and standard deviation of each cephalometric parameter are shown in Table 3. From Table 3, it is evident that the basal plane angle measurement had the least homogeneous distribution (CV = 30.14), while the R angle measurement had the most homogeneous distribution (CV = 6.91).

**Table 3:** Descriptive statistics of pooled group Assessment of variability of each parameter in entire sample statistically using coefficient of variance. Coefficient of variance (CV) was expressed in percentage. SN-GoGn: Steiner’s mandibular plane angle, FMA: Frankfort mandibular plane angle

	Mean	SD	CV (%)
SN-GoGn (deg)	29.22	7.20	24.64
FMA (deg)	24.35	7.02	28.85
Basal plane angle (deg)	22.93	6.91	30.14
Y-Axis (deg)	58.93	4.13	7.01
R Angle (deg)	73.5	5.08	6.91
DR Angle (deg)	31.82	3.55	11.15
Jarabak ratio	2.32	0.16	7.21
Facial height ratio	13.84	2.37	17.18

**Figure 6:**
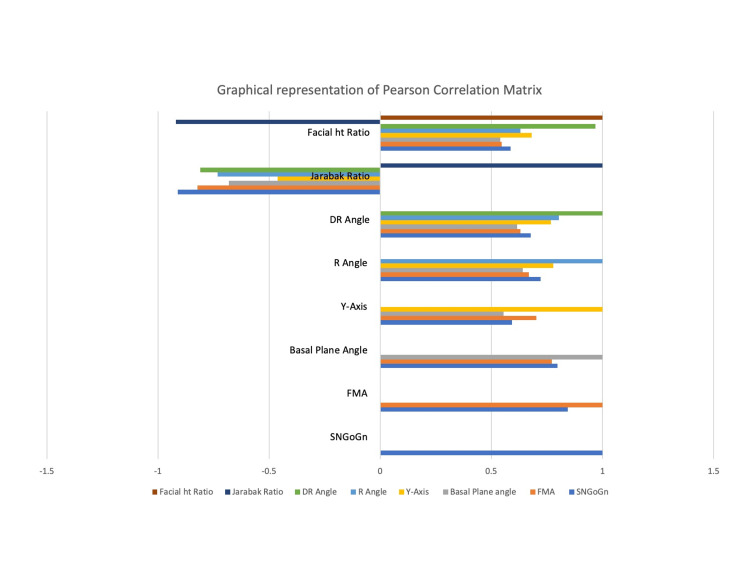
Graphical representation of correlation of the eight vertical parameters SN-GoGn: Steiner’s mandibular plane angle, FMA: Frankfort mandibular plane angle

The results showed a statistically significant and highly correlated relationship between the study's parameters used to assess the vertical jaw relationship, as shown in Table 4 and graphical representation in Figure 6. The correlation between the different skeletal analyses was ascertained through Pearson correlation. Facial height ratio and DR angle (r = 0.96), facial height ratio and Jarabak ratio (r = -0.91), SN-GoGn and FMA (r = 0.84), R angle and DR angle (r = 0.80), and DR angle and Jarabak ratio (r = -0.81) all showed a significant correlation. The Jarabak ratio and the facial height ratio, two linear measurements, were found to have a very strong correlation (r=-0.91) with one another. The remaining parameters showed a moderate correlation. Newer parameters, such as the R and DR angles, show a strong correlation (r = 0.80) with one another, but only a moderate correlation with the Y-axis, SN-GoGn, FMA, and basal plane angle. However, DR angle showed a strong relationship (r = 0.96) with linear parameters, namely the Jarabak ratio (r = -0.81) and the facial height ratio (r = 0.96). The Jarabak ratio and the Y-axis angle had the weakest (negative) correlation (r = -0.46).

**Table 4:** Correlation matrix for SN-GoGn, FMA, basal plane angle, Y-axis, R angle, DR angle, Jarabak ratio and facial height ratio Statistically Significant Correlation (P-value<0.001). Pearson correlation coefficient (r): weak correlation (0.01 < r < 0.5 or -0.5 < r < -0.01); moderate correlation (0.5 < r < 0.8 or -0.8 < r < -0.5); strong correlation (0.8 < r < 1 or -1 < r < -0.8). r > 0 implies positive correlation. r < 0 implies negative correlation. r = 0 implies no correlation. The null hypothesis (H0): The correlation between the two variables is zero. The alternative hypothesis: (Ha): The correlation between the two variables is not zero, e.g., there is a statistically significant correlation. If we use a significance level of α = .001, then we would reject the null hypothesis in this case if p-value < 0.001. Conclusions: The correlation coefficients are statistically significant. SN-GoGn: Steiner’s mandibular plane angle, FMA: Frankfort mandibular plane angle

	SN-GoGn (deg)	FMA (deg)	Basal Plane angle (deg)	Y-Axis (deg)	R Angle (deg)	DR Angle (deg)	Jarabak Ratio	Facial ht Ratio
SN-GoGn (deg)	1							
p-value								
FMA (deg)	0.843803997	1						
p-value	<<0.001							
Basal plane angle (deg)	0.79709876	0.771437656	1					
p-value	<<0.001	<<0.001						
Y-axis (deg)	0.59165797	0.70132424	0.553876948	1				
p-value	<<0.001	<<0.001	<<0.001					
R angle (deg)	0.721523465	0.667502628	0.641288719	0.777683386	1			
p-value	<<0.001	<<0.001	<<0.001	<<0.001				
DR angle (deg)	0.677701255	0.630957259	0.616397277	0.768466046	0.803082984	1		
p-value	<<0.001	<<0.001	<<0.001	<<0.001	<<0.001			
Jarabak ratio	-0.910506638	-0.823311922	-0.68065971	-0.462749254	-0.732895471	-0.810998636	1	
p-value	<<0.001	<<0.001	<<0.001	<<0.001	<<0.001	<<0.001		
Facial ht ratio	0.585924491	0.54571046	0.539469409	0.68187649	0.630140989	0.967833606	-0.919549184	1
p-value	<<0.001	<<0.001	<<0.001	<<0.001	<<0.001	<<0.001	<<0.001	

The current investigation produced an acceptable methodical error when evaluating linear measurements that were less than 0.5 mm and angular measurements on the lateral cephalograms were less than 0.5 degrees, according to Dahlberg's formula for method error calculation [10].

## Discussion

​​​Accurate evaluation of an individual's facial skeletal pattern in all three dimensions-transverse, vertical, and sagittal-is crucial for orthodontic diagnosis and treatment planning. By characterizing the variability in treatment planning, mechanics, and facial proportions, the vertical facial pattern plays a crucial role in orthodontics during the diagnosis and treatment planning processes.

According to Tweed the vertical growth pattern of the face can be linked to the stability of mandibular incisors following orthodontic treatment [4]. Given that the face stops growing vertically last, evaluating facial discrepancy in this dimension is crucial for both effective treatment planning and accurate diagnosis. It also plays a critical role in preventing relapse following correction of malocclusion.

A person's vertical growth pattern can be evaluated using a variety of skeletal metrics. Numerous times, disparate metrics yield contradictory findings, making it difficult to pinpoint a precise diagnosis [8]. In order to reduce the number of analyses required for diagnosis, this study concentrated on assessing the diagnostic accuracy of few select parameters.

Steiner's mandibular plane angle, or SN-GoGn, is a highly variable and unreliable metric to evaluate growth pattern, according to McNamara [11]. This could be caused by the fluctuating location of gnathion (Gn), which is reliant on the chin's spatial position. A study by Paranhos et al. [12] found that because Gn position varies with sagittal malocclusion, hence Y-axis was insufficient to evaluate vertical dysplasia. Schudy also found that the Y-axis was insufficient in another study [13]. According to a study by Asad et al. [14], the variance of the basal plane angle was high. This is because, depending on growth and dentoalveolar compensation, the palatal and mandibular planes have varying inclinations.

Out of all the linear measurements, the Jarabak ratio offers more insight into the growth pattern than the facial height ratio. The Jarabak ratio is more representative than the facial height ratio because unlike the latter it considers both the anterior and posterior facial height [15].

New parameters, the R angle and the DR angle, were developed in 2013 and 2017, respectively, in an effort to address the above-mentioned shortcomings. The intersection of the C-N and C-Me axes forms the R angle [16] at the condyle's centre (C). The R angle's mean value falls between 70.5^◦^ and 74.5^◦^. A horizontal growth pattern is represented by an angle less than 70.5^◦^, and a vertical growth pattern is represented by an angle more than 74.5^◦^.

Additionally, lines connecting Point M, the pre-maxilla centre, and Point G, the centre of the largest circle tangent to the internal inferior, anterior, and posterior surfaces of the mandibular symphysis, form the DR angle [17] at Point C, the condyle centre. A vertical growth pattern is indicated by the DR angle above 32.5°, an average growth pattern is indicated between 28.5 and 32.5°, and a horizontal growth pattern is indicated below 28.5°.

An individual's vertical growth pattern can be assessed using a variety of criteria. Along with the analyses that are frequently used to diagnose orthodontic problems and show a particular pattern of jaw growth in relation to the cranial base, the current study also included recently proposed parameters, like R angle and DR angle, to assess their validity in comparison to commonly used analyses.

A significant correlation was observed between all skeletal analyses in our study. Between SN-GoGn, FMA, R angle, DR angle, and Jarabak ratio, there was a strong correlation observed.

There was a moderate to weak correlation between the Y-axis, basal plane angle, and facial height ratio and other skeletal analyses. Our findings concur with those of other studies by Ahmed et al. [18] and Asad et al. [14].

We can infer from our study that newer parameters like R angle and DR angle can be used routinely to assess the growth pattern. However, parameters like the Y-axis and basal plane angle which are dependent on the spatial position of the chin and dentoalveolar compensation respectively can be excluded [19]. Among linear measurements, the facial height ratio only considers anterior facial height (upper and lower) whereas the Jarabak ratio includes the posterior facial height as well making it more representative so these parameters should be used in combination for more realistic diagnosis.

A possible limitation of this study could be the use of two-dimensional (lateral cephalogram) imaging rather than three-dimensional (3-D) imaging to assess the skeletal-jaw relationship, given the advancements in digital imaging techniques. Current research indicates that manual and digital lateral cephalograms are still relevant and reliable for scientific research, with the added benefit of a lower radiation dose, even though cone beam computed tomography (CBCT)-generated images are superior at evaluating skeletal jaw discrepancy [20,21].

## Conclusions

This study compared the diagnostic performance of several vertical dysplasia assessment parameters and suggested that, in order to obtain an accurate assessment of the vertical jaw relationship, a combination of different measurements should be used.

Among angular variables DR angle, R angle, SN-GoGn and FMA can be used and among linear variables Jarabak ratio could be used reliably to assess the growth pattern. Further studies should be conducted on a wider population of different race, ethnicity and demographics to validate the result of the present study.

## References

[1] Ricketts RM (1982). The biologic significance of the divine proportion and Fibonacci series. Am J Orthod.

[2] Ackerman JL, Proffit WR, Sarver DM, Ackerman MB, Kean MR (2007). Pitch, roll, and yaw: describing the spatial orientation of dentofacial traits. Am J Orthod Dentofacial Orthop.

[3] Nanda SK (1988). Patterns of vertical growth in the face. Am J Orthod Dentofac Orthop.

[4] Tweed CH (1946). The Frankfort-mandibular plane angle in orthodontic diagnosis, classification, treatment planning, and prognosis. Am J Orthod Oral Surg.

[5] Neger M (1951). The facial goniometer: an instrument for the direct measurement of the Frankfort-mandibular plane angle and the gonion angle. Angle Orthod.

[6] Steiner CC (1953). Cephalometrics for you and me. Am J Orthod.

[7] Downs WB (1948). Variations in facial relationships: their significance in treatment and prognosis. Am J Orthod.

[8] Bock JJ, Fuhrmann RA (2007). Evaluation of vertical parameters in cephalometry. J Orofac Orthop.

[9] Solow B, Tallgren A (1971). Natural head position in standing subjects. Acta Odontol Scand.

[10] Trpkova B, Major P (1997). Cephalometric landmarks identification and reproducibility: a meta analysis. Am J Orthod Dentofac Orthop.

[11] McNamara J (1984). A method of cephalometric evaluation. Am J Orthod.

[12] Paranhos LR, Brando TM, Kaieda AK, Ramos AL, Torres FC (2014). The inadequacy of the Y-axis of growth (SNGn) for the vertical pattern assessment in patients with sagittal discrepancies. J Contemp Dent Pract.

[13] Schudy F (1963). Vertical growth versus anteroposterior growth as related to function and treatment plane. Angle Orthod.

[14] Asad S, Naeem S (2009). Correlation between various vertical dysplasia assessment parameters. Pakistan Orthod J.

[15] Bahrou S (2014). Facial proportions in different mandibular rotations in class I individuals. Int Arab J Dent.

[16] Mohammed R, Mascarenhas R (2013). A new parameter for assessing vertical skeletal discrepancies: the R angle. Rev Latinoam Ortod.

[17] Lekhadia DR, Rai R, Hegde N, Hegde G, Sorake A, Kumar A (2015). Assessment of vertical skeletal patterns using a new cephalometric parameter: the Dhaval—Rohan angle. J Postgrad Med Educ Res.

[18] Ahmed M, Shaikh A, Fida M (2016). Diagnostic performance of various cephalometric parameters for the assessment of vertical growth pattern. Dental Press J Orthod.

[19] Ricketts RM (1961). Cephalometric analysis and synthesis. Angle Orthod.

[20] Ramírez Huerta JV, Oropeza Sosa JG, Flores Ledesma A (2015). Comparative study between cone-beam and digital lateral head film cephalometric measurements. Rev Mex Ortod.

[21] van Bunningen RH, Dijkstra PU, Dieters A, van der Meer WJ, Kuijpers-Jagtman AM, Ren Y (2022). Precision of orthodontic cephalometric measurements on ultra low dose-low dose CBCT reconstructed cephalograms. Clin Oral Investig.

